# Myasthenia Gravis Presenting as Bulbar Palsy

**DOI:** 10.7759/cureus.46082

**Published:** 2023-09-27

**Authors:** Dhruv Gosain, Tapas Das

**Affiliations:** 1 General Internal Medicine, Peterborough City Hospital, Peterborough, GBR; 2 Gastroenterology, Peterborough City Hospital, Peterborough, GBR

**Keywords:** thymoma, neuromuscular junction disorders, bulbar palsy, auto-immune diseases, myasthenia gravis (mg)

## Abstract

Myasthenia gravis is a rare autoimmune condition that affects postsynaptic cholinergic receptors, resulting in symptoms of muscular fatigue. Clinical signs could be subtle and variable, often leading to many differentials. This leads to inappropriate tests being performed and a delay in diagnosis. Although ocular signs are more common, it may rarely present as bulbar palsy. Our patient, in her 30s, was referred to the emergency department after six months of symptom onset when she was discovered to be at a high risk of silent aspiration. Her presentation was predominantly bulbar palsy, but after appropriate tests, she was eventually diagnosed with generalized myasthenia gravis with a concurrent thymoma. Her treatment included pyridostigmine, corticosteroid, and immunoglobulins, while a thymectomy was scheduled as a planned procedure. Prompt diagnosis and timely management can reduce morbidity and mortality in such cases.

## Introduction

Myasthenia gravis (MG) is an autoimmune condition that affects postsynaptic cholinergic receptors, manifesting as fatigable muscle weakness that improves with rest [1]. Although it can affect any muscle group, ocular symptoms are the typical presentations, seen as ptosis and diplopia. In 80% of patients, this weakness may then become generalized [2]. Subtle clinical findings and fluctuating nature of symptoms make MG difficult to diagnose. In a study, it was found that in 13% of patients, the diagnosis was delayed by five years, while 26% of patients had inappropriate and non-specific investigations performed prior to the actual diagnosis [3]. In addition to the ocular signs, bulbar symptoms such as slurred speech, dysphagia, and dysphonia can lead to a broad range of differential diagnoses [4].

We report a case of generalized MG that presented with predominantly bulbar symptoms. The diagnosis was made six months after symptom onset when she was found to have silent aspirations during swallowing. This case demonstrates the range of differential diagnoses due to the varied presenting symptoms and clinical findings and the need for an appropriate and timely referral to a specialist.

## Case presentation

A woman in her 30s was referred to the emergency department following a barium swallow test, which showed an uncoordinated pharyngeal swallow with a significant risk of silent aspiration.

Six months ago, she began experiencing weakness in her arms after exercising in the gym, which gradually worsened to involve her neck muscles as well. She noticed that it improved on the days she skipped strenuous exercises. This weakness gradually progressed, and she was unable to chew or close her jaw completely. Over a few months, she noticed a nasal tone in her voice and eventually difficulty swallowing, which led to a weight loss of about 5 kg. Her general practitioner found no neurological deficit on examination and hence referred her to the dentist who performed an X-ray of the mandible and an orthopantomogram, both of which were normal. She was then referred to the hospital because of her dysphagia and unremarkable neurological examination, where she saw the Ear-Nose-Throat (ENT) specialist. A flexible naso-endoscopy was performed, which showed some prominent lymphoid tissue in the tongue base with normal vocal cord mobility. Subsequently, a barium swallow was arranged, which revealed an uncoordinated pharyngeal swallow with a significant risk of silent aspiration.

Immediate referral to the emergency department was made where the medical teams’ examination revealed slight brisk reflexes in her right upper and lower limbs, bilateral facial nerve weakness with forehead sparing, an inability to close her eyes completely, and poor palatal movement. At the time of initial presentation, there was no ptosis or diplopia seen, and there was no reduction in the amplitude of movements of the proximal limbs. She denied peripheral muscle weakness, visual changes, headache, hearing loss, or any sensory deficit. With no past medical history of chronic disease, she also denied any regular consumption of alcohol or smoking. There was no family history of neurological conditions or autoimmune diseases. She was not on any regular medications, which could have precipitated MG.

The initial impression was bulbar palsy, with the differential diagnosis of motor neuron disease (MND), MG, and brainstem compression. Although she had brisk reflexes during the initial neurological examination, she lacked mixed upper and lower motor signs as seen in MND. A computed tomography (CT) scan of brain, spine, and blood samples for acetylcholinesterase antibodies was requested, along with other routine blood tests. Based on the above imaging studies, stroke and brainstem compression were ruled out; however, an incidental soft tissue mass of 4.5 × 2.5 cm was found in the mediastinum. This was first thought to be a mediastinal lymph node and raised suspicion of lymphoma when correlating it with the history of weight loss. Due to the risk of aspiration, the patient was kept nil by mouth, and a neurologist referral was made.

When examined by the neurologist on the third day of admission, the patient was found to have full eye movements. The left eyelid was found to be slightly ptotic on prolonged upward gaze. There was bilateral moderate facial weakness and nasal dysarthria but normal tongue movements. Shoulder abductions fatigued completely with less than 20 repetitions. The given history and findings were highly suggestive of generalized MG, and in this context, the mediastinal mass was most likely thought to be a thymoma, which was later confirmed by a magnetic resonance imaging (MRI) scan of the thorax.

Investigations

Since admission to the hospital, various blood tests were performed, as presented in Table 1.

**Table 1:** Blood tests on admission. AChR: acetylcholine receptor; MuSK: muscle-specific tyrosine kinase.

Blood test	Results	Reference range	Unit
White blood count	6.3	4.0-11.0	x 10^9 ^/ L
Hemoglobin	136	115-165	g/L
Platelet	411	150-400	x 10^9^ / L
Creatine kinase	53	25-200	U/L
C-reactive protein	18	<5	mg/L
Creatinine	54	45-84	μmol/L
Lactate dehydrogenase	173	<250	U/L
Thyroid-stimulating hormone	1.50	0.30 – 4.20	mU/L
Sodium	139	133 - 146	mmol/L
Potassium	4.4	3.5 – 5.3	mmol/L
AChR antibodies	>20	<0.50	nmol/L
MuSK antibodies	Negative	<0.05	nmol/L

The routine blood tests were not concerning, and the viral screen for hepatitis and HIV was negative. The results of acetylcholine receptor (AChR) antibodies were received on the fifth day of admission, and levels were >20 nmol/L (normal range <0.50 nmol/L). Muscle-specific tyrosine kinase (MuSK) antibodies were negative.

**Figure 1:**
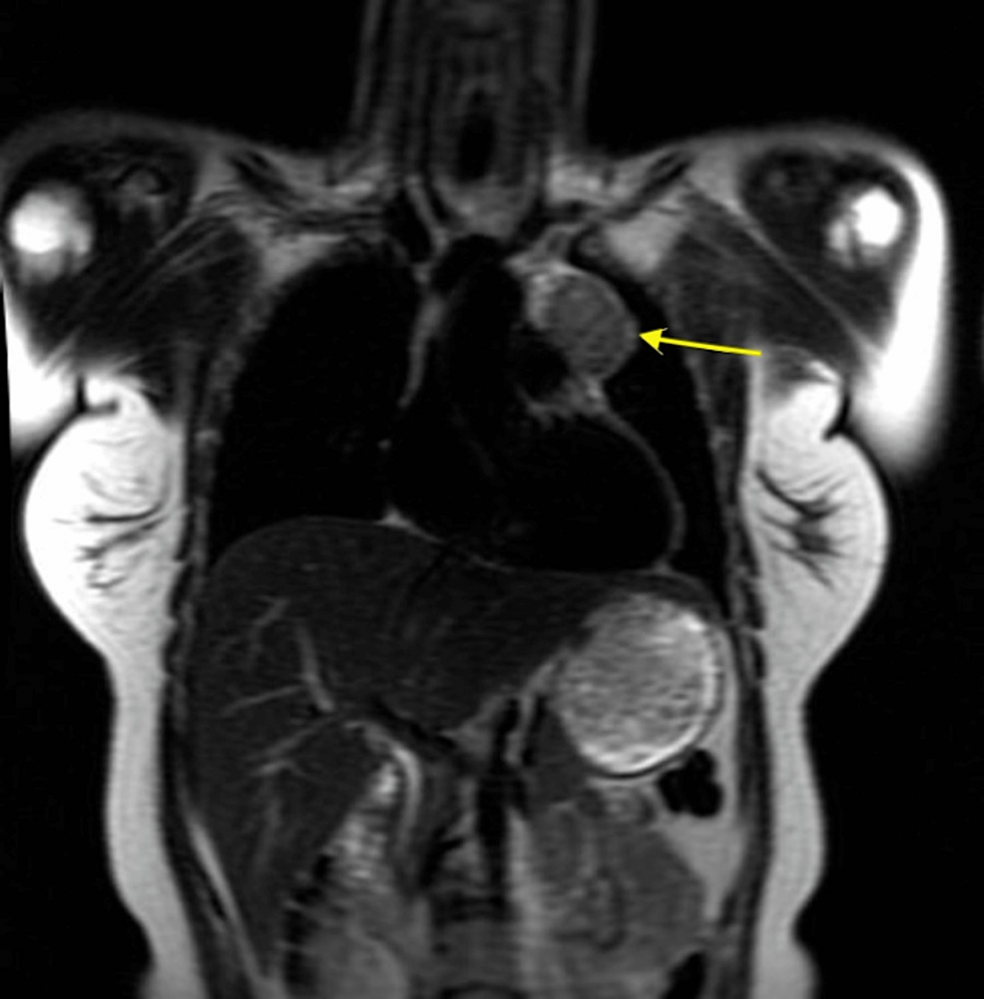
MRI thorax coronal view showing a thymoma. MRI: magnetic resonance imaging.

**Figure 2:**
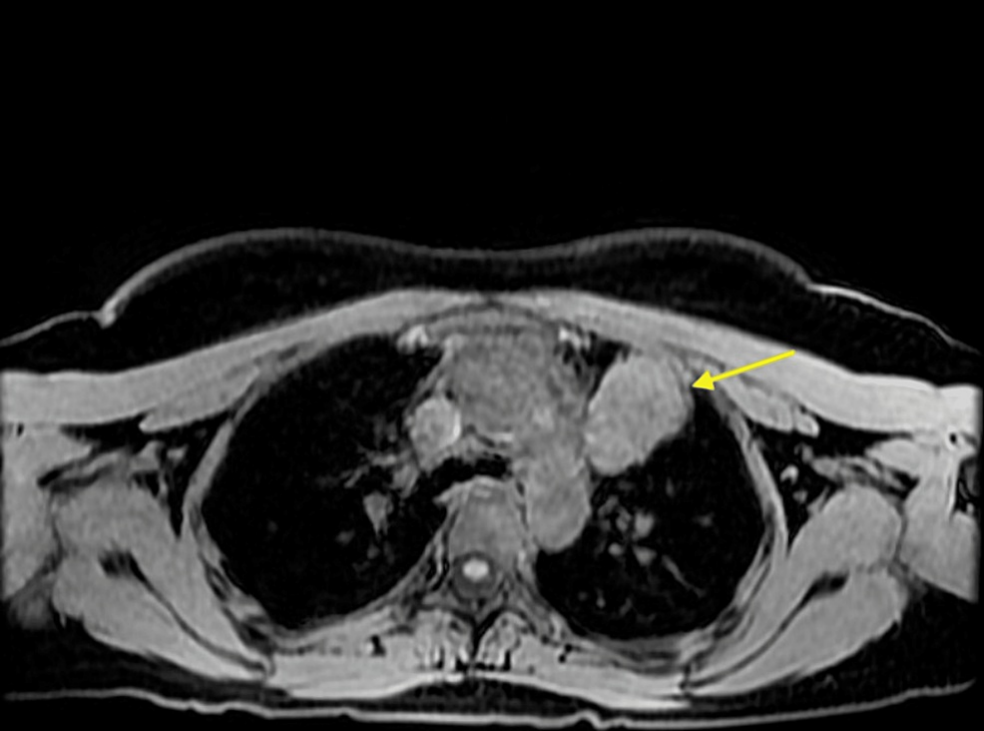
MRI thorax axial view showing a thymoma. MRI: magnetic resonance imaging.

Keeping in mind the differentials, multiple scans were performed. While the CT scan picked up the left anterior mediastinal mass, the MRI scan of thorax revealed a well-defined mass measuring 4.3 x 3.7 x 4.4 cm. It was of predominantly uniform intermediate T2 and intermediate T1 signal intensities, with a few small internal vessels. There appeared to be a thin septum posteriorly. The lesion demonstrated uniform mild contrast enhancement. The septum posteriorly was more suggestive of a thymoma rather than a lymphoma. There were no appreciable chemical shift artifacts, a feature suggestive of a thymoma rather than thymic hyperplasia (Figures 1, 2). 

Pulmonary function tests performed on the third day of admission and on the day of discharge were both in the normal range (Table 2).

**Table 2:** Pulmonary function tests on day 3 of admission and day of discharge. FVC: forced vital capacity; FEV1: forced expiratory volume in the first second.

Pulmonary function tests	Day 3 of admission	Day of discharge	Normal range
FVC	82.4%	87.7%	>80% of predicted value
FEV1	82.7 %	86.6 %	>80% of predicted value
FEV1/ FVC	0.83	0.82	>0.70
Vital capacity	3.67 L	3.70 L	3-5 L

Treatment

The patient was started on pyridostigmine 30 mg four times a day and propantheline 15 mg, as and when required for any gastrointestinal side effects. There was a significant improvement in her proximal muscle strength and jaw movements; however, symptoms such as hypernasality were still present. After 10 days, pyridostigmine was gradually increased to a daily dose of 180 mg, followed by 60 mg four times a day.

Due to the significant symptoms of severe dysphagia and dysphonia, intravenous immunoglobulins were administered at a dose of 1 g/kg body weight over four days. She was started on oral steroids at 10 mg of prednisolone, once a day for three days, followed by 20 mg for the next three days, and then at 25 mg daily dose.

On discussion with thoracic surgeons, there was no indication for a biopsy of the mediastinal mass, and an outpatient appointment was scheduled with them for assessing the thymoma and planning the surgery. With the symptomatic improvement, safe swallowing abilities, and normal pulmonary function tests, the patient was discharged. On outpatient appointments, she was clinically stable on the above steroid dose and was followed up by the surgeons. 

She later underwent a median sternotomy for resection of the anterior mediastinal mass, and the histology confirmed a WHO type AB invasive thymoma with TNM (Tumor, Node, Metastasis) stage pT1a pNX, V0, which did not require radiotherapy. 

## Discussion

MG is a rare autoimmune condition that particularly affects the postsynaptic cholinergic receptors and manifests as fatigue symptoms that improve with rest [5]. Studies recently conducted in Europe show the incidence rates of the disease to be between 4.1 and 30 cases per million person-years, while the incidence of generalized myasthenia ranges between 2.4 and 3.17 per million people annually [6,7]. While the majority of the patients exhibit autoantibodies against AChRs, a small percentage have antibodies against MuSK, low-density lipoprotein receptor-related protein 4 (Lrp4), or Agrin [8-11].

The involvement of the thymus gland in this disease is well known; however, the exact pathogenesis is still unclear. The AChR is expressed on myoid cells of this gland, and in patients with antibodies against AChRs, there is hyperplasia of the thymic medulla, with germinal centers surrounded by a T-cell zone. Seventy percent of MG patients have hyperplastic alterations (germinal centers), which show an active immune response, and 10% of patients have a thymoma. These are regions of lymphoid tissue where helper T-cells and B-cells work together to make antibodies. Hence, thymectomy remains an important element of treatment for such patients [12].

MG can be clinically classified into two categories: one with pure ocular symptoms and generalized myasthenia with mild, moderate, and severe symptoms [13]. Although it can cause substantial morbidity and even mortality, the condition is often curable. With prompt disease diagnosis and effective treatment, this can typically be avoided.

Treatment modalities include symptomatic relief with ACh inhibitors, such as pyridostigmine, as the initial line of treatment [14]. Depending on the severity of symptoms, immunomodulator therapies may be required. Patients with generalized MG with moderate symptoms who require long-term corticosteroids may require a thymectomy or second-line immunosuppressants for their steroid-sparing effects [14,15]. Although side effects restrict long-term use, oral steroids are usually the first line of immunosuppressive therapy; however, one must avoid high initial doses of prednisolone as this could possibly worsen symptoms [16]. The second-line immunosuppressive medications are azathioprine, tacrolimus, methotrexate, and mycophenolate mofetil [14]. Plasma exchange therapy and intravenous immunoglobulins may be used in refractory cases, in situations where rapid response is needed, like prior to surgery, or when the risk of myasthenia crisis is high [17].

In advanced cases, it is also important to perform pulmonary function tests to prevent progression to myasthenia crisis. Values defining myasthenia crisis are a vital capacity of less than 1 L (or <20-25 mL/kg) or a negative inspiratory force (NIF) of less than 20 cm H_2_O, both of which indicate significant respiratory weakness and the need for mechanical ventilation [18].

While the typical symptoms of myasthenia are well known, the broad range of presenting complaints raises various differentials. As in our patient, bulbar symptoms on presentation are only observed in 15% of cases [19]. Brisk reflexes must not be unequivocal evidence of an upper motor disorder, as it could also be physiological or associated with anxiety and even cervical spondylosis [20]. As seen in other case reports, myasthenia has been misdiagnosed as posterior circulation stroke, amyotrophic lateral sclerosis, velopharyngeal incompetence, myofascial pain syndrome, and even hysteria [19]. Inappropriate investigations are thus requested, and this leads to a delay in diagnosis. In a recent study, it was found that irrespective of the age of onset, the mean diagnostic delay for myasthenia was slightly over one year [20]. If mistreated, it can lead to the myasthenia crisis, which has a high mortality rate.

## Conclusions

The importance of careful history-taking is evident from our case. Even though the patient did not present with overtly specific neurological signs at various instances, a six-month history of fatigability from strenuous exercise should have raised suspicion early on. In this disease, clinical findings may be subtle but important, such as fatigability on repeated arm abductions, ptosis on prolonged upward gaze, and the presence of normal reflexes, tone, and power of the limbs. MG should also be considered in patients presenting with focal bulbar symptoms. Relevant blood investigations and imaging studies, along with timely referral to the neurologist, can lead to the appropriate diagnosis and management.
